# Impact of Acute and Chronic Stressors on the Morphofunctional Characteristics of Long Bones in Spontaneously Hypertensive Rats: A Pilot Study Using Histological and Microtomographic Analysis

**DOI:** 10.3390/biomedicines13071689

**Published:** 2025-07-10

**Authors:** Marina Ribeiro Paulini, Dimitrius Leonardo Pitol, Sara Feldman, Camila Aparecida Ribeiro, Daniela Vieira Buchaim, Rogerio Leone Buchaim, João Paulo Mardegan Issa

**Affiliations:** 1 Department of Basic and Oral Biology, School of Dentistry of Ribeirão Preto, University of São Paulo (FORP-USP), Ribeirão Preto 14040-904, Brazil; marina.paulini@usp.br (M.R.P.); dimitrius@forp.usp.br (D.L.P.); 2 LABOATEM—Laboratory of Ostearticular Biology and Tissue Engineering, School of Medicine, Rosario S2002, Argentina; saryfeldman@gmail.com; 3 Research Council, National Rosario University (CIUNR-CONICET), Rosario S2002, Argentina; 4 Medical School, University Center of Adamantina (FAI), Adamantina 17800-000, Brazil; camilaribeiro@fai.com.br (C.A.R.); danibuchaim@alumni.usp.br (D.V.B.); 5 Graduate Program in Anatomy of Domestic and Wild Animals, Faculty of Veterinary Medicine and Animal Science, University of São Paulo (FMVZ/USP), Sao Paulo 05508-270, Brazil; rogerio@fob.usp.br; 6 Postgraduate Department, Dentistry School, Faculty of the Midwest Paulista (FACOP), Piratininga 17499-010, Brazil; 7 Center for the Study of Venoms and Venomous Animals, São Paulo State University (CEVAP/UNESP), Botucatu 18619-002, Brazil; 8 Department of Biological Sciences, School of Dentistry of Bauru, University of São Paulo (FOB-USP), Bauru 17012-901, Brazil

**Keywords:** hypertension, stress, bone remodeling, spontaneously hypertensive rats, microtomography, histology, chronic stress, acute stress, bone metabolism, cardiovascular diseases

## Abstract

**Background/Objectives:** Hypertension is a major contributor to cardiovascular diseases and is often intensified by psychological stress, which can also affect bone metabolism. Although both conditions independently compromise bone health, their combined impact—particularly under acute and chronic stress—remains unclear. This pilot study aimed to assess the effects of such stressors on bone structure in spontaneously hypertensive rats (SHRs). **Methods:** Forty male rats, both normotensive and SHRs, were randomly assigned to control, acute stress, or chronic stress groups. Acute stress involves a single 2 h physical restraint. Chronic stress was induced over 10 days using alternating stressors: agitation, forced swimming, physical restraint, cold exposure, and water deprivation. Tibial bones were analyzed by microcomputed tomography (micro-CT), and histology was performed using Hematoxylin and Eosin and Masson’s Trichrome stains. **Results:** Micro-CT showed increased trabecular bone volume in normotensive rats under chronic stress, whereas SHRs displayed impaired remodeling under both stress types. Histological analysis revealed preserved connective tissue overall but evident changes in growth plate structure among stressed rats. SHRs exhibited exacerbated trabecular formation and cartilage abnormalities, including necrotic zones. **Conclusions:** Both acute and chronic stress, especially in the context of hypertension, negatively affect bone remodeling and maturation. Despite the absence of overt inflammation, structural bone changes were evident, indicating potential long-term risks. These findings highlight the importance of further studies on stress–hypertension interactions in bone health as well as the exploration of therapeutic approaches to mitigate skeletal damage under such conditions.

## 1. Introduction

Hypertension is one of the most prevalent cardiovascular diseases and a leading cause of mortality worldwide, commonly contributing to conditions such as stroke and acute myocardial infarction [1]. Numerous risk factors are associated with the development of hypertension, including obesity, smoking, alcohol consumption, family history, personality traits, and psychological factors such as stress. Both genetic predisposition and environmental elements—such as sedentary behavior and excessive sodium intake—are also frequently cited [2,3,4,5].

Despite the extensive literature on hypertension, relatively little attention has been devoted to the influence of emotional and psychological factors—such as stress, anxiety, hostility, and impulsivity—on blood pressure regulation [6,7,8]. It is hypothesized that stress triggers sympathetic nervous system activation, resulting in increased blood pressure [9,10,11]. Stressful experiences—ranging from interpersonal conflict to financial strain and social isolation—are known to provoke physiological and behavioral responses that can disrupt neuroendocrine and immune function, leading to diverse individual outcomes [12,13,14,15,16,17,18].

Bone tissue, similarly dynamic, undergoes continuous remodeling throughout life to maintain structural integrity and mineral homeostasis. Activities involving mechanical loading promote osteogenesis and help counteract bone loss [19,20]. Notably, hypertension has been linked to alterations in calcium metabolism and increased bone resorption, which may contribute to bone mineral density reduction in hypertensive individuals [21,22,23].

With the global rise in aging populations and lifestyle-related conditions—including physical inactivity, stress, and dietary imbalances—the prevalence of both hypertension and osteoporosis has escalated [24,25,26]. These disorders often coexist, influenced by a complex interplay of genetic and environmental factors [27,28].

Given the increasing prevalence of chronic diseases and emotional stress in the general population, there is a growing need for integrated research that examines physiological, morphological, and biochemical alterations in these conditions [29,30].

Therefore, this pilot study aimed to investigate the effects of acute and chronic stress, in combination with hypertension, on the skeletal system through histological assessment and microcomputed tomography (micro-CT) analysis. Our findings may provide further insight into the interactions between cardiovascular and skeletal health under stress-related conditions.

## 2. Materials and Methods

### 2.1. Ethical Aspects and Experimental Design

This study was approved by the Local Ethics Committee for the Use of Animals at the University of São Paulo/Brazil, at the School of Dentistry of Ribeirão Preto, registered under number 0027/2021R2. Male Hannover strain rats (*n* = 20) and SHRs (spontaneous hypertensive rats) (*n* = 20), weighing approximately 250 g at the start of the experiments, were used. The animals were obtained from the Central Animal Facility of the University of São Paulo, Ribeirão Preto campus, Brazil. The animals were kept in individual cages, in a room with a controlled temperature (24 ± 1 °C), with a 12 h light/dark cycle (light cycle starting at 7:00 AM), and had ad libitum access to water and food. The experimental protocols were conducted in a quiet room during the morning and in the same location (same laboratory) to minimize variations. Animal care, including manual recording of body weight, food intake, water consumption, and cage alterations, occurred daily at 8:00 AM during the light cycle. The rats were left undisturbed throughout the entire dark cycle.

The animals were randomly divided into three main groups: the control group with no stress (NS); the group with acute stress (A); and the group with chronic varied stress (C). Each group was subdivided into normal rats and spontaneously hypertensive rats. Groups A and C were further subdivided into control and stress-exposed groups, totaling 40 animals.

### 2.2. Acute Stress Protocol

Sixteen animals, both normal and SHRs, were subjected to a protocol involving a single episode of stress (acute stress for 2 h—physical restraint). The animals were placed in a metal box measuring 15 cm in length and 5 cm in diameter, with adequate ventilation throughout its length. The end of the box was closed, and the animals were in a state of physical restraint for 2 h.

### 2.3. Chronic Varied Stress Protocol

**Figure 1:**
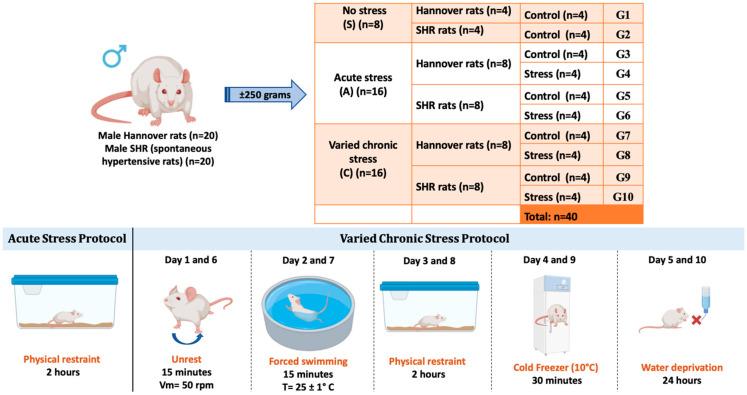
Division of experimental groups with acute and chronic varied stress protocols. G1: NS-Control (normal rats); G2: NS-Control (SHRs); G3: A-Control (normal rats); G4: A-Acute Stress (normal rats); G5: A-Control (SHRs); G6: A-Acute Stress (SHRs); G7: C-Control (normal rats); G8: C-Chronic stress (normal rats); G9: C-Control (SHRs); G10: C-Chronic stress (SHRs).

Chronic varied stress is an effective protocol as it simulates frequent daily conditions to which individuals are exposed. Several studies in the literature use the chronic stress protocol for different analyses, using different stressors, generally 1 to 3 times a day, for varied periods. A chronic varied stress protocol was carried out according to a previous study by our research group [31,32,33]. In this protocol, five distinct forms of stress were implemented over a total period of 10 days. The stressful situations were initiated in the morning, according to the following description: Days 01 and 06: agitation. The rats were individually placed in a plastic box on a shaker table for 15 min. The average rotation speed was 50 rpm. Days 02 and 07: forced swimming. The rats swam for 15 min in a circular plastic container, 54 cm deep and 47 cm in diameter, filled with water to a depth of 40 cm, preventing contact with the upper or lower edges. The water temperature was controlled and maintained at 25 ± 1 °C. Days 03 and 08: physical restraint. The animals were placed in a metal box measuring 15 cm in length and 5 cm in diameter, with adequate ventilation throughout its length. The end of the box was closed, and the animals were physically restrained for two hours, limiting their movement. Days 04 and 09: cold stress. The rats, in individual plastic boxes, were exposed to hypothermia in the freezer (10 °C) for a period of 30 min. Days 05 and 10: water deprivation. The water source was removed for a period of 24 h (Figure 1).

### 2.4. Tibia Collection and Microtomographic Evaluation

At the end of the experiment, the animals were euthanized, having been previously anesthetized with 4% xylazine (14 mg/kg) and 10% ketamine (100 mg/kg), via intraperitoneal injection, and were subjected to euthanasia by conscious decapitation 24 h after the last stress exposure. In rats subjected to acute stress, euthanasia was performed immediately after the stress. Subsequently, the right and left tibias of each animal were removed, dissected, and fixed in 10% formaldehyde phosphate buffer (pH 7.4) for 48 h.

All samples were scanned using micro-CT (Skyscan model 1172, Bruker-Micro-CT^®^, Kontich, Belgium). The region of interest (ROI) started at the marginal crest of the tibia and extended 2 mm, corresponding to 21 slices. The device was set to 70 kV and 142 uA. A filter (0.5) was used, and the sample was rotated 180 degrees with a rotation step of 0.5, generating an acquisition time of 41 min per sample. NRecon software program (version 1.7.4.6, Bruker, Kontich, Belgium) was used to reconstruct the three-dimensional images. Subsequently, the images were aligned in the coronal, transaxial, and sagittal planes using the DataViewer program v3.2.0.

### 2.5. Histological Analysis

The samples were removed, dissected, and maintained in 10% EDTA solution until complete decalcification. The samples were left in 10% buffered formaldehyde for 24 to 48 h, then transferred to 10% EDTA under constant agitation for decalcification until complete decalcification. The specimens were processed histologically for inclusion in paraffin enriched with Histosec polymer (Merck KGaA^®^, Darmstadt, Germany). Coronal sections of 5 μm thickness were obtained and stained with Hematoxylin and Eosin (HE) and Masson’s Trichrome. All histological slides were coded according to the experimental groups, and an experienced examiner—blinded to the group assignments—performed the qualitative evaluations to mitigate potential observer bias. All images were captured under identical camera settings, including white balance, gain, and exposure. The exposure time was selected to limit the occurrence of saturated intensity pixels. A total area of 144 × 106 pixels^2^ was evaluated for each sample. Virtual microscopy, data management, and image analysis were performed with the aid of a digital camera attached to the Zeiss AxioImager Z2 light microscope (Oberkochen, Germany), with original magnifications of ×20 and ×40. Subsequently, the digital images were analyzed using AxioVision 4.8 software (Carl Zeiss, Oberkochen, Germany).

### 2.6. Statistical Analysis

The percentage of newly formed bone assessed through micro-CT was first tested for normal distribution using the Kolmogorov–Smirnov test and for homogeneity of variances using Bartlett’s test. Following this, one-way ANOVA was applied, and differences between group means were examined with Tukey’s post hoc test. The statistical analyses were performed in GraphPad Prism (version 8.0, GraphPad^®^ Software, La Jolla, CA, USA), adopting a significance threshold of 5%.

## 3. Results

The descriptive statistical analyses and their normality, for all the variables included in the objectives of this study, showed them to be normal (*p* > 0.05), according to the significance of the Shapiro–Wilk test.

### 3.1. Linear Measurements of the Tibia in Micro-CT Imagens

**Figure 2:**
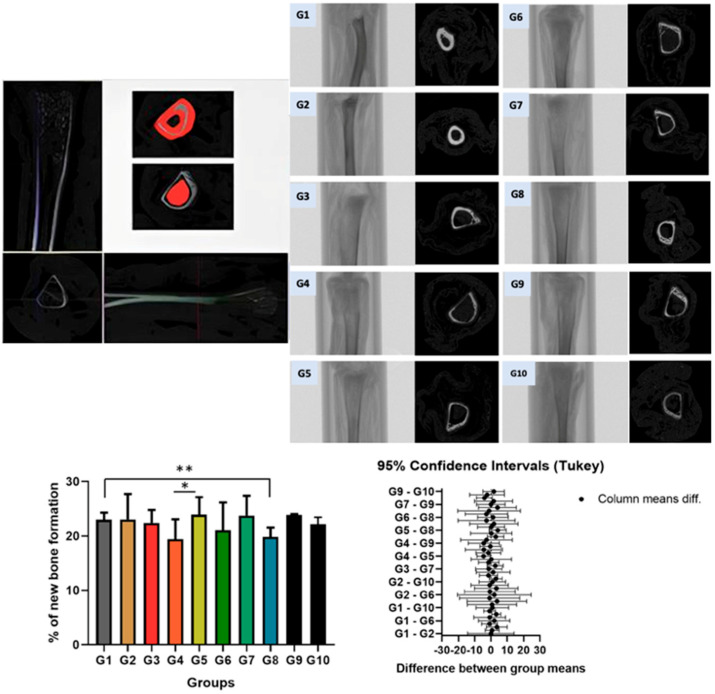
Micro-CT results of tibial bone. A significant difference was observed between group G1: NS-Control (normal rats) and G8: C-Chronic stress (normal rats), as well as between G4: A-Acute stress (normal rats) and G5: A-Control (SHRs) (spontaneously hypertensive rat control). No significant statistical difference was found between the other groups. * Statistical difference between groups G4 and G5. ** Statistical difference between groups G1 and G8.

Three-dimensional (3D) and two-dimensional (2D) microtomographic images of the tibial bone in groups G1 to G10 are shown in Figure 2. The analysis focused on the proximal region of the bone. The reference point was determined at the end of the growth plate, “Top selection,” and then 2 mm down, corresponding to 21 slices, to reach “Bottom selection.” The bone area in the trabecular and cortical regions was delineated starting from the coronal section. The percentage of bone volume was quantified between the groups. A significant difference was observed between group G1: NS-Control (normal rats) and G8: C-Chronic stress (normal rats), as well as between G4: A-Acute stress (normal rats) and G5: A-Control (SHRs) (spontaneously hypertensive rat control). No significant statistical difference was found between the other groups (Table 1, Table 2 and Table 3).

### 3.2. Descriptive Histology

Qualitative analysis of the histological sections was performed using Hematoxylin and Eosin (HE) and Masson’s Trichrome staining, both applied to assess overall tissue morphology. Overall, all experimental groups exhibited preserved connective tissue architecture, with no evidence of acute or chronic inflammatory infiltrates. However, distinct morphological alterations were observed in groups G4 and G8, particularly concerning the structural organization of the growth plate, suggesting potential effects of the experimental conditions on bone maturation and remodeling mechanisms in this region.

**Figure 3:**
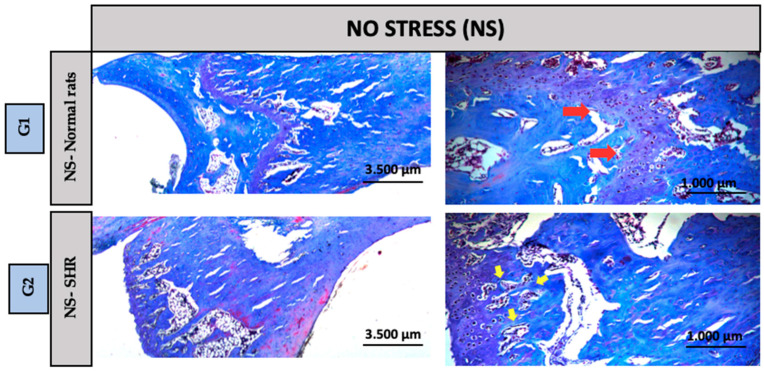
Groups G1: NS-Control (normal rats) and G2: NS-Control (SHRs): In the distal region of the tibial bone, a 2.5× and 10× increase. Red arrow = growth plate or cartilage. Yellow arrow = bone trabeculae.

**Figure 4:**
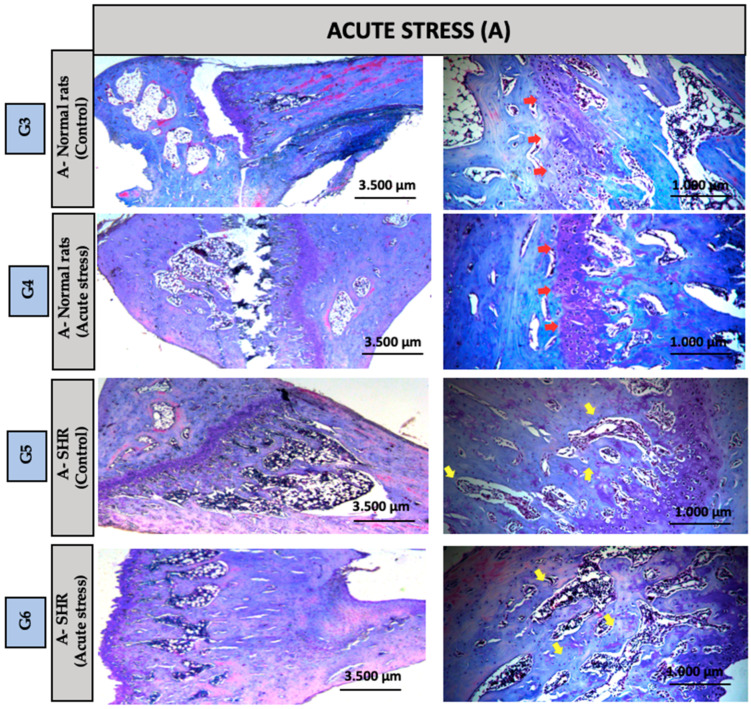
Groups G3: A-Control (normal rats) and G4: A-Acute Stress (normal rats): In the distal region of the tibial bone, a 2.5× and 10× increase. Red arrow = growth plate or cartilage. Groups G5: A-Control (SHRs) and G6: A-Acute Stress (SHRs): In the distal region of the tibial bone, a 2.5× and 10× increase. Yellow arrow = bone trabeculae plate.

**Figure 5:**
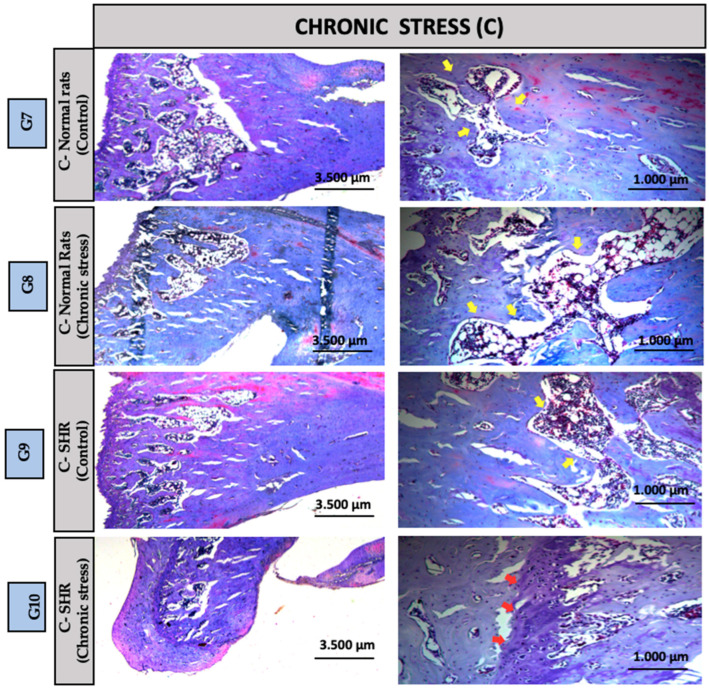
Groups G7 to G10 (Chronic Stress): In the distal region of the tibial bone, a 2.5× and 10× increase. Red arrow = growth plate or cartilage. Yellow arrow = bone trabeculae plate.

Groups G1 and G2 (Control): In the distal region of the tibia, Group G1: NS-Control (normal rats) exhibited a clearly preserved growth plate (red arrow), whereas Group G2: NS-Control (SHRs) exhibited a more organized bone structure with greater evidence of bone trabeculae (yellow arrow). Despite these structural differences, the overall tissue morphology remained preserved in both groups (Figure 3). Groups G3 and G4 (Acute Stress): In the distal region of the tibia, Group G3: A-Control (normal rats) exhibited a morphological pattern similar to that observed in Group G1, with a clearly visible growth plate (red arrow). Group G4: A-Acute Stress (normal rats) despite exposure to the stressor, maintained histological features comparable to those of Group G3 A-Control (normal rats), suggesting that the acute stress episode did not induce evident structural alterations in bone architecture (Figure 4). Groups G5: A-Control (SHRs) and G6: A-Acute Stress (SHRs): In the distal region of the tibial bone, we can observe a more organized bone with greater evidence of bone trabeculae, suggesting that hypertension exacerbated the effects of acute stress on extracellular matrix remodeling (Figure 4). Chronic Stress Groups (G7 to G10): G7: C-Control (normal rats)—A morphological pattern similar to G1: NS-Control (normal rats) was observed. G8: C-Chronic stress (normal rats)—This group exhibited the highest content and organization of bone trabeculae, with the presence of red-stained tissue indicating mature bone. MicroCT results revealed a significant difference compared to G1: NS-Control (normal rats), suggesting a pronounced adaptive response to prolonged stress. G9: C-Control (SHRs) and G10: C-Chronic stress-SHRs—Both groups showed a morphology similar to the control group G2: NS-Control (SHRs), without indicating alterations due to chronic stress associated with hypertension (Figure 5).

**Figure 6:**
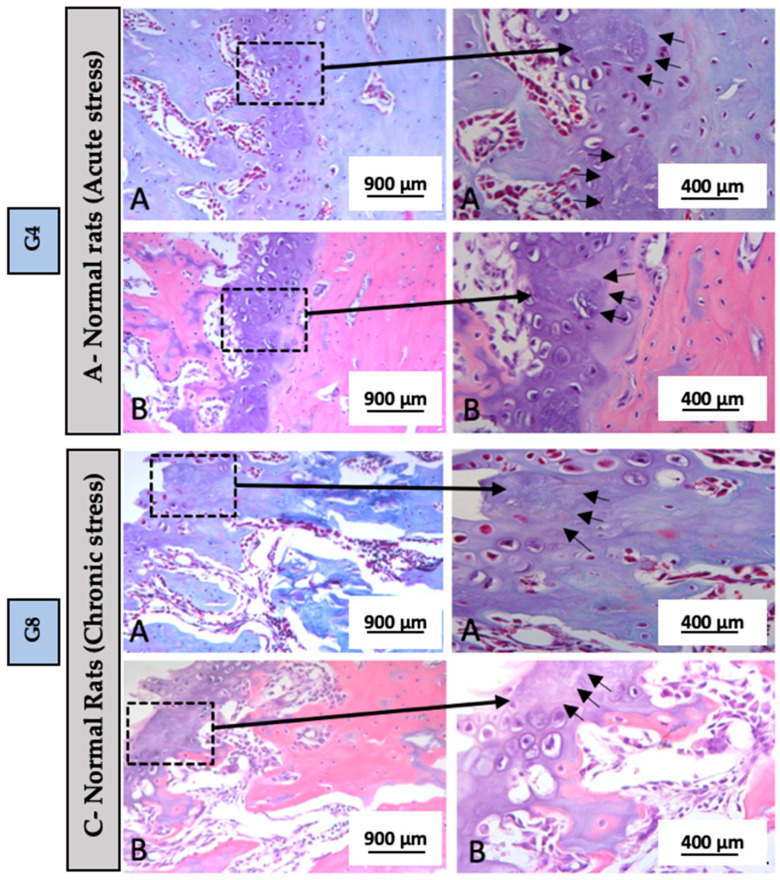
(**A**) Hematoxylin and Eosin staining and (**B**) Masson’s Trichrome staining, at 20× (900 µm) and 40× (400 µm) magnification. Black arrows = alteration in cartilage morphology with the presence of necrosis in the growth plate in groups G4: A-Acute Stress (normal rats) and G8: C-Chronic stress (normal rats).

It is important to highlight groups G4 and G8 because they were the groups that presented significant results in micro-CT. When evaluating the micro-CT results, it was observed that Group G4: A-Acute Stress (normal rats) showed the lowest percentage of bone formation, followed by Group G8: C-Chronic stress (normal rats). Due to this significant difference between the groups, only Groups G4 and G8 were selected for histological imaging in Figure 6, in order to better illustrate the findings. In these images, we were able to visually observe greater tissue evidence through Hematoxylin and Eosin staining, as well as Masson’s Trichrome. Additionally, in the growth plate, alterations in cartilage morphology were identified, including areas of necrosis indicated by black arrows (Figure 6). Areas of necrosis in bone tissue may suggest avascular necrosis, a condition in which parts of the bone die due to lack of blood supply. This can be caused by trauma, excessive alcohol use, certain diseases or treatments, and can lead to pain, stiffness and eventually collapse of the bone. In the case of this study, it may suggest stress.

## 4. Discussion

The primary objective of this study was to evaluate the impact of acute and chronic stress, in association with hypertension, on bone metabolism in rats. Bone remodeling is a dynamic process involving the balance between bone modeling and resorption. Our observations indicate that both acute and chronic stress significantly affect bone architecture, with notable differences between normotensive and hypertensive groups.

Different skeletal sites may respond variably to physiological or pathological stressors, including emotional stress and hypertension. Studies have demonstrated the sensitivity of the tibial bone to chronic psychosocial stress and its suitability as a reliable experimental model in bone research. Foertsch et al. [34] demonstrated that chronic psychosocial stress significantly impairs tibial growth and ossification in adolescent mice, indicating the responsiveness of the tibia to systemic stress signals. Haffner-Luntzer et al. [35] further confirmed that the tibia is susceptible to impaired healing and endochondral ossification under chronic stress conditions mediated by β-adrenergic signaling. Despite this support, we acknowledge that limiting our analyses to the tibia may not fully capture skeletal heterogeneity.

The interpretation of chronic stress as an “adaptive” phenomenon in groups subjected to this condition remains speculative and lacks a clear mechanistic basis. Although the concept of adaptation to stress is widely discussed in the literature, the chronic effects of stress on bone metabolism may actually reflect a pathological imbalance in bone turnover, characterized by increased resorption and dysregulated formation, which can lead to conditions such as early osteosclerosis [36,37]. Studies indicate that chronic stress activates the hypothalamic–pituitary–adrenal (HPA) axis, increasing the release of glucocorticoids that, in excess, have deleterious effects on osteoblasts and promote cell apoptosis, contributing to pathological bone remodeling [38,39]. Furthermore, changes in bone microarchitecture observed in chronic stress models can be interpreted as early signs of osteosclerosis, a condition characterized by abnormally increased bone density but with compromised bone quality [40]. Therefore, it is essential to consider these alternative interpretations for a more complete understanding of the effects of chronic stress on bone metabolism.

Recent studies indicate that chronic stress can influence bone remodeling through modulation of the RANKL/OPG pathways, which are essential for osteoclast differentiation and activation. Glucocorticoid signaling also plays a crucial role, affecting osteoblast and osteoclast function and interfering with bone homeostasis. The integration of these molecular pathways may contribute to a broader understanding of how chronic stress affects bone metabolism. However, the current evidence does not fully support a definitive mechanistic interpretation, and the assumption that the observed changes are adaptive remains speculative in some instances. Therefore, caution is warranted when interpreting such effects, as they may also reflect pathological alterations in bone turnover rather than physiological adaptation [41,42,43,44,45,46,47].

In rats subjected to acute stress (G4 and G5), an increase in trabecular density was observed, especially in hypertensive rats (G5). While this may suggest a possible stress-induced modulation of bone metabolism, potentially mediated by sympathetic nervous system activation and modulation of the hypothalamic–pituitary–adrenal axis [48,49], these findings should be interpreted conservatively. Although data on acute stress and bone metabolism remain limited, studies involving chronic stress indicate that exposure to stress can influence bone remodeling. For instance, Azuma et al. [50] highlighted the impact of chronic psychological stress on bone metabolism, emphasizing the role of the HPA axis and glucocorticoid activity. Similarly, Henneicke et al. [51] demonstrated that chronic mild stress induces bone loss through a glucocorticoid-dependent mechanism specifically in osteoblasts. These findings suggest that stress-induced changes in bone metabolism may involve either compensatory or deleterious mechanisms, depending on the nature, duration, and intensity of the stressor, rather than reflecting a uniformly adaptive process.

In rats exposed to chronic stress (G8), a more pronounced reorganization of trabecular bone architecture was observed, suggesting structural adaptation. Chronic stress has been shown to affect the balance between osteoblasts and osteoclasts [52], although in this study, bone formation remained relatively stable, indicating adaptive rather than degenerative changes [53]. Hypertension, especially in groups G5 and G10, also influenced bone remodeling. It may disrupt the balance between bone formation and resorption, amplifying the effects of stress [54]. For example, the increased trabecular density in G5 may reflect an adaptive response to the combined effects of hypertension and stress [55].

Although previous studies have examined the isolated effects of stress and hypertension on bone [34], their interaction remains less understood. Our findings highlight the importance of managing both conditions to preserve skeletal integrity [56]. MicroCT analysis showed that G8 (normotensive with chronic stress) had the highest trabecular bone volume, supporting evidence that chronic stress can trigger adaptive bone formation [57,58]. In contrast, hypertensive rats (G9 and G10) did not show significant bone changes, aligning with studies indicating that hypertension alone may not significantly alter bone structure over short periods [29].

Group G4: A-Acute Stress (normal rats) showed reduced bone volume compared to G1 (control), suggesting an unfavorable response to acute stress, consistent with findings that acute stress elevates cortisol levels, impairing bone homeostasis [53,59]. However, G5 (hypertensive with acute stress) showed no additional changes, indicating that short-term combined exposure may not further alter bone microarchitecture [35].

Histological analysis using Hematoxylin and Eosin and Masson’s Trichrome stains revealed significant changes in the growth plate, notably in G4 and G8, with necrotic areas corresponding to reduced bone volume and mineralization seen in MicroCT [35]. These findings suggest that acute stress, especially when combined with hypertension, may impair growth plate integrity and bone formation [60,61].

Despite some limitations—such as the use of SHRs and the short experimental period—this study provides relevant clinical insights. Longer exposure periods might reveal more extensive remodeling or degenerative changes [62,63]. The limited timeframe may also have prevented detection of cumulative effects linked to chronic stress and hypertension [64,65,66]. Preliminary findings from the qualitative, non-quantitative histological analysis are presented, with the understanding that these will be complemented by additional analyses, including quantitative histological and immunohistochemical assessments. Limiting the analyses to the tibia may not fully capture skeletal heterogeneity. Therefore, this is considered a limitation, and we acknowledge the need for future studies to include additional skeletal sites, such as the femur or vertebrae, to broaden the interpretation of skeletal responses under the conditions studied. Although the experimental design was based on previously validated models cited in the literature, it recognizes the importance of including physiological validation. Thus, biomarkers such as serum corticosterone levels, heart rate variability, or behavioral assessments were not included in the present study. These measures would have strengthened the confirmation of a physiological response to stress. Future studies are planned to incorporate these parameters in order to provide a more comprehensive assessment of stress induction and its systemic effects. It is worth highlighting the importance of quantitative histomorphometric analysis, such as trabecular thickness, chondrocyte count and inflammatory cell count. However, in the present study, a qualitative histological evaluation was conducted based on standardized staining protocols and performed by experienced observers to evaluate morphological changes in bone and cartilaginous structures.

These results emphasize the clinical relevance of monitoring bone health in individuals exposed to psychosocial stress and hypertension. Future studies should investigate molecular pathways, inflammatory mediators, and therapeutic strategies—both pharmacological and behavioral—to mitigate bone loss and promote skeletal health.

## 5. Conclusions

This study explored the potential relationships between stress, hypertension, and bone remodeling, with observations of some alterations in bone formation and cartilage morphology in groups exposed to chronic stress and hypertension. However, given the limited and somewhat inconsistent evidence, conclusions regarding the negative impact of stress on bone maturation should be made with caution. Additionally, while no clear signs of inflammation were noted, the absence of specific inflammatory marker analyses limits definitive statements on inflammatory involvement. Limitations such as the duration of exposure and the use of an animal model must also be considered when interpreting these findings. Future research with longer experimental periods and comprehensive assessments—including inflammatory markers and potential therapeutic interventions—are needed to clarify these relationships and to support development of more effective prevention and treatment strategies for bone-related diseases, especially in individuals experiencing chronic stress and cardiovascular conditions.

## Figures and Tables

**Table 1:** Percentage of new bone formation measured on micro-CT.

	G1	G2	G3	G4	G5	G6	G7	G8	G9	G10
Mean ± SD	23.02 ± 1.26 **	23.06 ± 4.66	22.41 ± 2.39	19.42 ± 3.65 *	23.93 ± 3.21 *	21.06 ± 5.01	23.78 ± 3.63	19.83 ± 1.73 **	23.85 ± 0.19	22.19 ± 2.81

Mean ± standard deviation. ANOVA and Tukey’s post-test, *p* < 0.05. The presence of a single asterisk (*) indicates a significant difference between groups G4 and G5, as well as two asterisks (**) between G1 and G8.

**Table 2:** ANOVA table.

	SS	DF	MS	F (DFn, DFd)
Treatment (between columns)	121.2	9	13.47	F (1.745, 6.978) = 1.329 F (4, 36) = 1.124
Individual (between rows)	45.57	4	11.39	
Residual (random)	364.8	36	10.13	
Total	531.6	49		

SS = Sum of Squares; DF = Degrees of Freedom; MS = Mean Square; F = Statistic; DFn = Degrees of Freedom numerator; DFd = Degrees of Freedom denominator.

**Table 3:** Micro-Computed Tomography (micro-CT) Analysis: Morphometric parameters obtained by micro-CT of the tibiae. The parameters analyzed include tissue volume (TV), bone volume (BV), percentage of bone volume (BV/TV), tissue surface (TS), bone surface (BS), intersection surface (i.S), bone surface to volume ratio (BS/BV), bone surface density (BS/TV) and trabecular pattern factor (Tb.Pf). The values are expressed in the respective units of measurement indicated. NS: No Stress; AS: Acute Stress; CS: Chronic Stress; SHR: Spontaneous Hypertensive Rats.

			G1	G2	G3	G4	G5	G6	G7	G8	G9	G10
			NS	NS	AS	AS	AS	AS	CS	CS	CS	CS
			Normal rats	SHRs	Normal rats	Normal rats	SHRs	SHRs	Normal rats	Normal rats	SHRs	SHRs
Description	Abbreviation	Unit	Control	Control	Control	Acute Stress	Control	Acute Stress	Control	Chronic Stress	Control	Chronic Stress
Tissue volume	TV	mm^3^	12.733.594	12.789.941	131.448.460	15.228.645	124.989.966	13.912.630	1.245.267	1.482.602	1.241.090	13.474.538
Bone volume	BV	mm^3^	293.806	295.706	294.576	295.588	298.976	293.189	296.706	294.312	296.888	299.654
Percent bone volume	BV/TV	%	23.0175	23.065	22.41	19.4175	23.925	21.0625	23.775	19.835	23.85	22.19
Tissue surface	TS	mm^2^	4.993.918	3.831.324	4.778.871	4.593.316	3.279.641	4.546.161	2.957.578	4.232.392	4.501.669	3.974.099
Bone surface	BS	mm^2^	4.451.017	2.971.198	3.846.521	3.455.365	2.914.445	3.792.720	2.434.846	3.828.748	4.733.449	3.709.413
Intersection surface	i.S	mm^2^	255.385	718.400	188.105	203.964	235.366	229.826	198.548	222.050	210.603	194.604
Bone surface/volume ratio	BS/BV	1/mm	1.521.402	1.495.429	1.716.925	1.588.229	1.480.302	1.597.593	1.597.775	1.367.958	1.711.046	1.543.679
Bone surface density	BS/TV	1/mm	338.386	348.372	381.087	345.576	409.473	364.583	454.487	354.622	357.023	337.703
Trabecular pattern factor	Tb.Pf	1/mm	239.666	534.249	379.008	364.828	275.185	296.569	307.904	219.751	182.072	209.109

## Data Availability

The original contributions presented in this study are included in the article. Further inquiries can be directed at the corresponding author.

## References

[1] Dzau V.J., Hodgkinson C.P. (2024). Precision Hypertension. Hypertension.

[2] Ondimu D.O., Kikuvi G.M., Otieno W.N. (2019). Risk Factors for Hypertension Among Young Adults (18–35) Years Attending in Tenwek Mission Hospital, Bomet County, Kenya in 2018. Pan Afr. Med. J..

[3] Nwoke O.C., Nubila N.I., Ekowo O.E., Nwoke N.C., Okafor E.N., Anakwue R.C. (2024). Prevalence of Prehypertension, Hypertension, and Its Determinants Among Young Adults in Enugu State, Nigeria. Niger. Med. J..

[4] Wyszyńska J., Łuszczki E., Sobek G., Mazur A., Dereń K. (2023). Association and Risk Factors for Hypertension and Dyslipidemia in Young Adults from Poland. Int. J. Environ. Res. Public Health.

[5] Vo H.K., Nguyen D.V., Vu T.T., Tran H.B., Nguyen H.T.T. (2023). Prevalence and Risk Factors of Prehypertension/Hypertension Among Freshman Students from the Vietnam National University: A Cross-Sectional Study. BMC Public Health.

[6] Liu M.Y., Li N., Li W.A., Khan H. (2017). Association Between Psychosocial Stress and Hypertension: A Systematic Review and Meta-Analysis. Neurol. Res..

[7] Kluknavsky M., Balis P., Liskova S., Micurova A., Skratek M., Manka J., Bernatova I. (2024). Dimethyl Fumarate Prevents the Development of Chronic Social Stress-Induced Hypertension in Borderline Hypertensive Rats. Antioxidants.

[8] Wang Y., Ye C., Kong L., Zheng J., Xu M., Xu Y., Li M., Zhao Z., Lu J., Chen Y. (2023). Independent Associations of Education, Intelligence, and Cognition with Hypertension and the Mediating Effects of Cardiometabolic Risk Factors: A Mendelian Randomization Study. Hypertension.

[9] Franco C., Sciatti E., Favero G., Bonomini F., Vizzardi E., Rezzani R. (2022). Essential Hypertension and Oxidative Stress: Novel Future Perspectives. Int. J. Mol. Sci..

[10] Ojangba T., Boamah S., Miao Y., Guo X., Fen Y., Agboyibor C., Yuan J., Dong W. (2023). Comprehensive Effects of Lifestyle Reform, Adherence, and Related Factors on Hypertension Control: A Review. J. Clin. Hypertens..

[11] Wildenauer A., Maurer L.F., Rötzer L., Eggert T., Schöbel C. (2024). The Effects of a Digital Lifestyle Intervention in Patients with Hypertension: Results of a Pilot Randomized Controlled Trial. J. Clin. Hypertens..

[12] Muddle S., Jones B., Taylor G., Jacobsen P. (2022). A Systematic Review and Meta-Analysis of the Association Between Emotional Stress Reactivity and Psychosis. Early Interv. Psychiatry.

[13] Jiménez-Ortiz J.L., Islas-Valle R.M., Jiménez-Ortiz J.D., Pérez-Lizárraga E., Hernández-García M.E., González-Salazar F. (2019). Emotional Exhaustion, Burnout, and Perceived Stress in Dental Students. J. Int. Med. Res..

[14] Bajaña L.A.C., Campos Lascano L., Jaramillo Castellon L., Cevallos C.C., Cevallos-Pozo G., Ron B.V., e Silva F.F.V., Perez-Sayans M., Fiorillo L. (2023). The Prevalence of the Burnout Syndrome and Factors Associated in the Students of Dentistry in Integral Clinic: A Cross-Sectional Study. Int. J. Dent..

[15] Minshew L.M., Bensky H.P., Zeeman J.M. (2023). There’s No Time for No Stress! Exploring the Relationship Between Pharmacy Student Stress and Time Use. BMC Med. Educ..

[16] Kim Y., Choi Y., Kim H. (2022). Positive Effects on Emotional Stress and Sleep Quality of Forest Healing Program for Exhausted Medical Workers During the COVID-19 Outbreak. Int. J. Environ. Res. Public Health.

[17] Kweon J., Kim Y., Choi H., Im W., Kim H. (2024). Enhancing Sleep and Reducing Occupational Stress Through Forest Therapy: A Comparative Study Across Job Groups. Psychiatry Investig..

[18] Kane H.S., Wiley J.F., Dunkel Schetter C., Robles T.F. (2019). The Effects of Interpersonal Emotional Expression, Partner Responsiveness, and Emotional Approach Coping on Stress Responses. Emotion.

[19] Hart N.H., Newton R.U., Tan J., Rantalainen T., Chivers P., Siafarikas A., Nimphius S. (2020). Biological Basis of Bone Strength: Anatomy, Physiology and Measurement. J. Musculoskelet. Neuronal Interact..

[20] Yang N., Liu Y. (2021). The Role of the Immune Microenvironment in Bone Regeneration. Int. J. Med. Sci..

[21] Zhai X.L., Tan M.Y., Wang G.P., Zhu S.X., Shu Q.C. (2023). The Association Between Dietary Approaches to Stop Hypertension Diet and Bone Mineral Density in US Adults: Evidence from the National Health and Nutrition Examination Survey (2011–2018). Sci. Rep..

[22] Yuan M., Li Q., Yang C., Zhi L., Zhuang W., Xu X.S., Tao F. (2023). Waist-to-Height Ratio Is a Stronger Mediator in the Association Between DASH Diet and Hypertension: Potential Micro/Macro Nutrients Intake Pathways. Nutrients.

[23] Tan M.Y., Zhu S.X., Wang G.P., Liu Z.X. (2024). Impact of Metabolic Syndrome on Bone Mineral Density in Men Over 50 and Postmenopausal Women According to U.S. Survey Results. Sci. Rep..

[24] Fischer V., Haffner-Luntzer M. (2022). Interaction Between Bone and Immune Cells: Implications for Postmenopausal Osteoporosis. Semin. Cell Dev. Biol..

[25] Zhou P., Lu K., Li C., Xu M.-Z., Ye Y.-W., Shan H.-Q., Yin Y. (2024). Association Between Systemic Inflammatory Response Index and Bone Turnover Markers in Chinese Patients with Osteoporotic Fractures: A Retrospective Cross-Sectional Study. Front. Med..

[26] Wu D., Cline-Smith A., Shashkova E., Perla A., Katyal A., Aurora R. (2021). T-Cell Mediated Inflammation in Postmenopausal Osteoporosis. Front. Immunol..

[27] Nakagami H., Morishita R. (2013). Hypertension and osteoporosis. Clin. Calcium.

[28] Huang Y., Ye J. (2024). Association Between Hypertension and Osteoporosis: A Population-Based Cross-Sectional Study. BMC Musculoskelet. Disord..

[29] Paulini M.R., Aimone M., Feldman S., Buchaim D.V., Buchaim R.L., Issa J.P.M. (2025). Relationship of Chronic Stress and Hypertension with Bone Resorption. J. Funct. Morphol. Kinesiol..

[30] Schemenz V., Scoppola E., Zaslansky P., Fratzl P. (2024). Bone Strength and Residual Compressive Stress in Apatite Crystals. J. Struct. Biol..

[31] Loyola B.M., Nascimento G.C., Fernández R.A., Iyomasa D.M., Pereira Y.C.L., Leite-Panissi C.R.A., Issa J.P.M., Iyomasa M.M. (2016). Chronic Stress Effects in Contralateral Medial Pterygoid Muscle of Rats with Occlusion Alteration. Physiol. Behav..

[32] Fernández R.A.R., Pereira Y.C.L., Iyomasa D.M., Calzzani R.A., Leite-Panissi C.R.A., Iyomasa M.M., Nascimento G.C. (2018). Metabolic and Vascular Pattern in Medial Pterygoid Muscle is Altered by Chronic Stress in an Animal Model of Hypodontia. Physiol. Behav..

[33] Pereira Y.C.L., Nascimento G.C., Iyomasa D.M., Fernández R.A.R., Calzzani R.A., Leite-Panissi C.R.A., Novaes P.D., Iyomasa M.M. (2019). Exodontia-Induced Muscular Hypofunction by Itself or Associated to Chronic Stress Impairs Masseter Muscle Morphology and Its Mitochondrial Function. Microsc. Res. Tech..

[34] Foertsch S., Haffner-Luntzer M., Kroner J., Gross F., Kaiser K., Erber M., Reber S.O., Ignatius A. (2017). Chronic psychosocial stress disturbs long-bone growth in adolescent mice. Dis. Model. Mech..

[35] Haffner-Luntzer M., Foertsch S., Fischer V., Prystaz K., Tschaffon M., Mödinger Y., Bahney C.S., Marcucio R.S., Miclau T., Ignatius A. (2019). Chronic psychosocial stress compromises the immune response and endochondral ossification during bone fracture healing via β-AR signaling. Proc. Natl. Acad. Sci. USA.

[36] Sapolsky R.M. (1996). Why stress is bad for your brain. Science.

[37] Manolagas S.C. (2000). Birth and death of bone cells: Basic regulatory mechanisms and implications for the pathogenesis and treatment of osteoporosis. Endocr. Rev..

[38] Weinstein R.S. (2010). Glucocorticoid-induced osteoporosis: Mechanisms and clinical implications. Endocrinol. Metab. Clin. N. Am..

[39] Hardy R.S., Zhou H., Seibel M., Cooper M.S. (2018). Glucocorticoids and Bone: Consequences of Endogenous and Exogenous Excess and Replacement Therapy. Endocr. Rev..

[40] Khosla S. (2013). Pathogenesis of osteoporosis. Mayo Clin. Proc..

[41] Weinstein R.S. (2012). Glucocorticoid-induced osteoporosis and osteonecrosis. Endocrinol. Metab. Clin. N. Am..

[42] Scheller E.L., Song J., Dishowitz M.I., Soki F.N., Hankenson K.D., Krebsbach P.H. (2013). Prolonged treatment with dexamethasone causes bone fragility by impacting osteoblast function and apoptosis. Bone.

[43] Lane N.E., Yao W. (2008). Glucocorticoid-induced osteoporosis: New insights into the pathophysiology and treatments. Curr. Osteoporos. Rep..

[44] O’Brien C.A., Jia D., Plotkin L.I., Bellido T., Powers C.C., Stewart S.A., Manolagas S.C., Weinstein R.S. (2004). Glucocorticoids act directly on osteoblasts and osteocytes to induce their apoptosis and reduce bone formation and strength. Endocrinology.

[45] Brennan-Speranza T.C., Henneicke H., Gasparini S.J., Blankenstein K.I., Heinevetter U., Cogger V.C., Svistounov D., Zhang Y., Cooney G.J., Buttgereit F. (2012). Osteoblasts mediate the adverse effects of glucocorticoids on fuel metabolism. J. Clin. Investig..

[46] Makino A., Nakamura Y., Okada H., Morikawa K., Ogata E., Yamamoto M. (2001). Glucocorticoid enhances receptor activator of NF-kappaB ligand expression and suppresses osteoprotegerin in human osteoblastic Saos-2 cells. Endocrinology.

[47] Plotkin L.I., Bellido T. (2016). Osteocytic signalling pathways as therapeutic targets for bone fragility. Nat. Rev. Endocrinol..

[48] Berger J.M., Singh P., Khrimian L., Morgan D.A., Chowdhury S., Arteaga-Solis E., Horvath T.L., Domingos A.I., Marsland A.L., Yadav V.K. (2019). Mediation of the Acute Stress Response by the Skeleton. Cell Metab..

[49] Xu H.K., Liu J.X., Zhou Z.K., Zheng C.X., Sui B.D., Yuan Y., Kong L., Jin Y., Chen J. (2024). Osteoporosis under psychological stress: Mechanisms and therapeutics. Life Med..

[50] Azuma K., Adachi Y., Hayashi H., Kubo K.Y. (2015). Chronic Psychological Stress as a Risk Factor of Osteoporosis. J. UOEH.

[51] Henneicke H., Li J., Kim S., Gasparini S.J., Seibel M.J., Zhou H. (2017). Chronic Mild Stress Causes Bone Loss via an Osteoblast-Specific Glucocorticoid-Dependent Mechanism. Endocrinology.

[52] Wippert P.M., Rector M., Kuhn G., Wuertz-Kozak K. (2017). Stress and Alterations in Bones: An Interdisciplinary Perspective. Front. Endocrinol..

[53] Tiyasatkulkovit W., Promruk W., Rojviriya C., Pakawanit P., Chaimongkolnukul K., Kengkoom K., Teerapornpuntakit J., Panupinthu N., Charoenphandhu N. (2019). Impairment of Bone Microstructure and Upregulation of Osteoclastogenic Markers in Spontaneously Hypertensive Rats. Sci. Rep..

[54] Nascimento R.M., Silva A.B., Lima R.M. (2024). Relationship of Chronic Stress and Hypertension with Bone Resorption. J. Funct. Morphol. Kinesiol..

[55] Ye Z., Lu H., Liu P. (2017). Association Between Essential Hypertension and Bone Mineral Density: A Systematic Review and Meta-Analysis. Oncotarget.

[56] Sato H., Richardson D., Cregor M., Davis H.M., Au E.D., McAndrews K., Zimmers T.A., Organ J.M., Peacock M., Plotkin L.I. (2017). Glucocorticoids Induce Bone and Muscle Atrophy by Tissue-Specific Mechanisms Upstream of E3 Ubiquitin Ligases. Endocrinology.

[57] Lima R.M., Souza P.L., Marques F.A., Fernandes D.B., Ribeiro T.C., Cardoso M.F., Nascimento W.R., Castro L.F., Barros G.M., Silva H.L. (2021). Combined Effects of Hypertension and Chronic Stress on Bone Microarchitecture in Rats. Bone Rep..

[58] Bonnet N., Ferrari S.L. (2010). Chronic psychosocial stress disturbs long-bone growth in adolescent mice. Dis. Model. Mech..

[59] Singewald G.M., Nguyen N.K., Neumann I.D., Singewald N., Reber S.O. (2009). Effect of Chronic Psychosocial Stress-Induced by Subordinate Colony (CSC) Housing on Brain Neuronal Activity Patterns in Mice. Stress.

[60] Ning B., Londono I., Laporte C., Villemure I. (2022). Validation of an in vivo micro-CT-based method to quantify longitudinal bone growth of pubertal rats. Bone.

[61] Mustafy T., Benoit A., Londono I., Moldovan F., Villemure I. (2018). Can repeated in vivo micro-CT irradiation during adolescence alter bone microstructure, histomorphometry and longitudinal growth in a rodent model?. PLoS ONE.

[62] Coleman R.M., Phillips J.E., Lin A., Schwartz Z., Boyan B.D., Guldberg R.E. (2010). Characterization of a small animal growth plate injury model using microcomputed tomography. Bone.

[63] Pereira A.C., Fernandes R.G., Carvalho Y.R., Balducci I., Faig-Leite H. (2007). Bone healing in drill hole defects in spontaneously hypertensive male and female rats’ femurs. A histological and histometric study. Arq. Bras. Cardiol..

[64] Bastos M.F., Brilhante F.V., Bezerra J.P., Silva C.A., Duarte P.M. (2010). Trabecular bone area and bone healing in spontaneously hypertensive rats: A histometric study. Braz. Oral Res..

[65] Tiyasatkulkovit W., Aksornthong S., Adulyaritthikul P., Upanan P., Wongdee K., Aeimlapa R., Teerapornpuntakit J., Rojviriya C., Panupinthu N., Charoenphandhu N. (2021). Excessive salt consumption causes systemic calcium mishandling and worsens microarchitecture and strength of long bones in rats. Sci. Rep..

[66] Sampath T.K., Simic P., Sendak R., Draca N., E Bowe A., O’Brien S., Schiavi S.C., McPherson J.M., Vukicevic S. (2007). Thyroid-stimulating hormone restores bone volume, microarchitecture, and strength in aged ovariectomized rats. J. Bone Miner. Res..

